# Reliability of Bolton analysis evaluation in tridimensional virtual models

**DOI:** 10.1590/2177-6709.20.5.072-077.oar

**Published:** 2015

**Authors:** Marianna Mendonca Brandão, Marcio Costal Sobral, Carlos Jorge Vogel

**Affiliations:** 1 Postgraduate student in Orthodontics and Facial Orthopedics, Universidade Federal da Bahia (UFBA), Salvador, Bahia, Brazil; 2 Professor, Universidade Federal da Bahia (UFBA), Postgraduate Program, Salvador, Bahia, Brazil; 3 PhD in Orthodontics, Universidade de São Paulo (USP), São Paulo, São Paulo, Brazil

**Keywords:** Computer-assisted diagnosis, Dental casts, Tridimensional imaging

## Abstract

**Objective:**

The present study aimed at evaluating the reliability of Bolton analysis in tridimensional virtual models, comparing it with the manual method carried out with dental casts.

**Methods:**

The present investigation was performed using 56 pairs of dental casts produced from the dental arches of patients in perfect conditions and randomly selected from Universidade Federal da Bahia, School of Dentistry, Orthodontics Postgraduate Program. Manual measurements were obtained with the aid of a digital Cen-Tech 4"^(r)^ caliper (Harpor Freight Tools, Calabasas, CA, USA). Subsequently, samples were digitized on 3Shape^(r) ^R-700T scanner (Copenhagen, Denmark) and digital measures were obtained by Ortho Analyzer software.

**Results:**

Data were subject to statistical analysis and results revealed that there were no statistically significant differences between measurements with *p*-values equal to *p* = 0.173 and *p*= 0.239 for total and anterior proportions, respectively.

**Conclusion:**

Based on these findings, it is possible to deduce that Bolton analysis performed on tridimensional virtual models is as reliable as measurements obtained from dental casts with satisfactory agreement.

## Abstract (pt)

**Objetivo:**

o presente estudo teve como objetivo avaliar a confiabilidade da análise de Bolton em modelos virtuais tridimensionais, comparando-a com a realizada em modelos de gesso.

**Métodos:**

foram utilizados 56 pares de modelos de gesso das arcadas dentárias de pacientes oriundos do Curso de Especialização em Ortodontia da Faculdade de Odontologia da Universidade Federal da Bahia, escolhidos aleatoriamente e em perfeito estado. Medidas manuais foram obtidas utilizando-se o paquímetro digital Cen-Tech^(r)^ 4" (Harpor Freight Tools, Calabasas, CA, EUA). Em seguida, os mesmos foram digitalizados pelo *scanner* R-700^TM^(3Shape^(r)^, Copenhagen, Dinamarca) e, por meio do programa Ortho Analyzer^TM^, da mesma marca, foram obtidas as medidas digitais.

**Resultados:**

os dados foram submetidos a testes estatísticos e os resultados demonstraram que não houve diferença estatisticamente significativa nos dois tipos de medições com valores de* p* = 0,173 e * p* = 0,239, respectivamente, para as proporções total e anterior.

**Conclusão:**

com base nesses achados, pode-se inferir que a análise de Bolton realizada em modelos virtuais tridimensionais é tão confiável quanto a obtida em modelos de gesso, apresentando uma concordância satisfatória.

## INTRODUCTION

When identifying patients' dental and bone problems, orthodontists rely on clinical findings, which are associated with radiographs, photographs and dental casts, to determine the most adequate treatment plan necessary to resolve each unique case.^1^

Dental casts allow malocclusions to be assessed tridimensionally and constitute one of the most important elements of diagnosis; thus, they are considered the "gold standard" in Orthodontics.^1^^-^ 4 Dental casts reproductions have acceptable reliability and enable complete assessment of patient's malocclusion, including shape and symmetry of dental arches and palate, individual dental positions, curves of Spee and Wilson, relationship between molars and canines, axial tipping of teeth, Bolton analysis, overbite and overjet, among other features.^5^^,^ 6

Correct overbite and overjet, as well as an adequate relationship between molars and canines result from the ideal proportional sum of mesiodistal diameters of both maxillary and mandibular teeth, among other aspects. The importance of proportionality for the orthodontist is obvious during the final phases of treatment. Minor discrepancies are insignificant from a clinical point of view; however, major discrepancies result in additional treatment challenges, requiring additional corrective treatment and/or compensations that were initially unplanned.^5^^,^ 7 ^,^ 8

In this context, a variety of methods have been developed to analyze discrepancy. The method proposed by Bolton in 1958 has become one of the most reliable methods, mainly due to its ease of execution and application.^9^^,^ 10 Bolton analysis is a valuable tool that is able to identify disagreement in tooth size between maxillary and mandibular teeth, which could negatively affect a correct dental relationship, highly desired during orthodontic treatment.^11^

When applying the formulas proposed by Bolton, if total proportion exceeds 91.3%, discrepancy corresponds to excess dental structure in the lower arch; whereas if proportion is lower than 91.3%, excess will be seen in the upper arch. If proportion in the anterior region exceeds 77.2%, excess dental structure will be in the lower arch; whereas if proportion is lower than 77.2%, it will be seen in the upper arch.^9^

Traditionally, Bolton indexes are measured manually with the aid of a bow divider or a caliper in dental casts.^7^ Nevertheless, with significant technological development, many orthodontists use computers and digitized orthodontic records to aid diagnosis and treatment planning.^4^^,^ 12 The use of scanned dental casts was announced by the orthodontic industry as the newest component of totally digitized records.^13^

The motivation for using digital models arose from the disadvantages of using dental casts, including the following: need for proper storage places, resulting in greater need for space in the office; risk of breaking which would cause permanent destruction of patient's records; duplication of casts in order to communicate with other dentists and specialists; increased hours of laboratory work and associated costs.^1^^,^ 4 ^,^ 6

Digital models and tridimensional technology minimize many of the previously mentioned problems, while providing the orthodontist with standard routine data, such as tooth size, overbite, overjet, Bolton and cast discrepancy, symmetry and shape of arches, intensity of the curves of Spee and Wilson, among others.^12^^,^ 14 ^,^ 15

However, as it is the case of any new method, it is necessary to assess the reliability of measurements taken with digital models, and correlating those results with the traditional dental cast method. Thus, the aim of this study is to assess the reliability of Bolton analysis performed on tridimensional virtual models, and compare those findings with the traditional dental cast method.

## MATERIAL AND METHODS

This is an experimental study that used dental casts taken from the dental arches of adult individuals. Initially, measurement taking was carried out by hand on dental casts, followed by digitization and digital measurement taking for comparison. The study was approved by Universidade Federal da Bahia Institutional Review Board (UFBA Protocol. #718.989/2014).

Initially, a total of 56 dental casts produced from the dental arches of patients treated and randomly selected from Universidade Federal da Bahia, School of Dentistry, Orthodontics Postgraduate Program were used. Dental casts were considered to have been perfectly preserved, with permanent teeth completely erupted and without the need for second and third molars.

Sample size calculation was performed by means of Epi Info software(version 6.0), using an expected difference of 0.09%, with test power of 80% and alpha level of 5%. Sample size (n) was determined at 56.

Direct measurements were taken on the dental casts (T_1_), followed by digitization. They corresponded to the largest mesiodistal width of all permanent teeth: first molars, pre-molars, canines and incisors. Measurement taking was performed by a single operator, previously trained. After 15 and 30 days (T_2_ and T_3_), 20% of casts were measured again, so as to confirm reproducibility.

Analysis of dental casts by means of the traditional method was performed with the aid of a digital caliper Cen-Tech 4" (Harpor Freight Tools, Calabasas, CA, USA), with precision of 0.01 mm. The caliper was placed on the buccal surface of teeth, starting with first molars, followed by second pre-molars, first pre-molars, canines and incisors on both upper and lower arches. Anterior and total proportion of mesiodistal sizes was calculated by summing teeth size up and determining the matching index.

For computer analysis, the models were digitized with a 3Shape**(r)** R-700(tm) scanner (Copenhagen, Denmark) that uses a non-destructive scanning method. The scanner consists of a platform to support the model, a laser and two high-resolution digital cameras used to capture the images. To ensure complete coverage of the object shape, the platform can be manipulated, so as to allow a double image to be captured.

Before digitization, the scanner was calibrated once a day, following the manufacturer's recommendations. The process began by appropriately positioning the model to be digitized onto the machine platform, so that the laser beam could map the desired profile.

For the digitization process, images were processed by 3Shape^(r)^ Ortho Impression(tm) software (Copenhagen, Denmark). After patient data had been recorded, digitization began. During this process, the laser captured images at specific dental cast locations, thereby producing a final virtual image. Image shape is a result of the organization of points in triangular shape. The image file was saved in DICOM format (Digital Imaging and Communications in Medicine).

Based on the digital images obtained, the digital models were manipulated by 3Shape^(r)^ Ortho Analyzer(tm) software (Copenhagen, Denmark).

**Figure 1:**
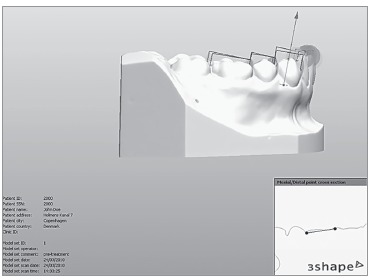
- Measurement of mesiodistal sizes by the digital method.

**Figure 2:**
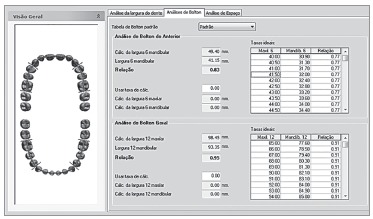
- Report of Bolton analysis by the digital method.

Digital measurements were taken by initially marking, from the buccal surface of teeth, the mesial and distal contact of first right maxillary molar, followed by second right maxillary pre-molar and all remaining teeth, until a complete set of measurements was obtained (Fig 1). The same approach was repeated on the lower arch. Thus, the software automatically generated the mesiodistal size of each tooth and the result of Bolton analysis (Fig 2).

The values obtained by both manual and digital measurement techniques were compiled in a MS Excel spreadsheet and statistically assessed by SPPS v. 15 software and MedCalc 9. Kolmogorov-Smirnov statistical test was performed to assess normality of data, thereby confirming the hypothesis of normal distribution of data.

Descriptive analysis (mean and standard deviation) was performed. To investigate the reliability of measurements found by the different techniques, Students t-test for paired samples was implemented. Lin's concordance coefficient (r) was used to determine whether measurements deviated significantly from perfect agreement. Excellent agreement was determined as r > 0.90, whereas satisfactory r ranged between 0.60 and 0.9, and unsatisfactory r was < 0.6. Significance level was set at 95% and results were descriptively presented in comparative tables generated on MS Word.

## RESULTS

The reproducibility of measurements obtained by means of the different methods were analyzed by Kappa test, with significance level set at 95%. No statistically significant differences were found between T_1_, T_2_ and T_3_, with Kappa = 0.9.

**Figure 3:**
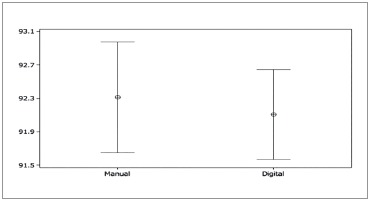
- Graphic comparing measurements of total proportion obtained by the manual and digital methods.

**Figure 4:**
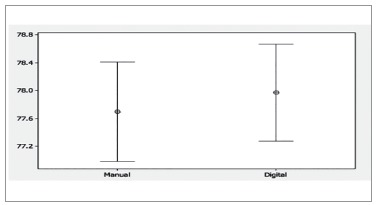
- Graphic comparing measurements of anterior proportion obtained with the manual and digital methods.

Student's t-test for paired samples was used to assess whether or not measurements presented any significant differences. Confidence interval was 95%. There were no statistically significant differences between measurements for any measurement approach, with *p* = 0.173 and *p* = 0.239 for total and anterior proportions, respectively (Figs 3 and 4).

**Figure 5:**
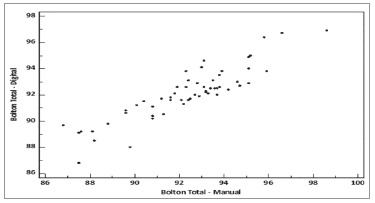
- Graphic of reproducibility of total proportion obtained with the manual and digital methods.

**Figure 6:**
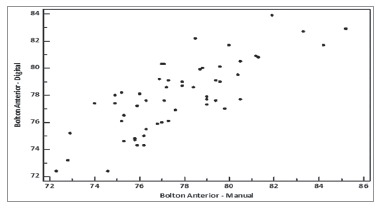
- Graphic of reproducibility of anterior proportion obtained with the manual and digital methods.

Measurement reproducibility assessed by means of Lin's concordance revealed that total proportion had r = 0.8715, with a confidence interval ranging from 0.7998 to 0.9187; whereas anterior proportion had r = 0.7785, with confidence interval ranging from 0.6506 to 0.8634 (Figs 5and 6). According to Lin, those values suggest that the digital method had satisfactory agreement, both in total and e anterior proportions.^16^

## DISCUSSION

With advances in technology, the use of digitized orthodontic records is becoming more and more common in clinical practice. Thus, it is necessary to test the effectiveness of this new digital method of which objective is to assist the orthodontist in visualizing, measuring and analyzing models, as well as in reaching diagnosis and treatment plan.

The present study found no statistically significant differences when Student's t-test was performed for paired samples to compare digitally or manually-obtained measurement methods, with *p* = 0.173 and *p* = 0.239 for total and anterior proportions, respectively. These results are similar to those obtained by Tomasseti et al,^12^ Paredes, Gandia and Cibrian,^17^Stevens et al^18^ and Mullen et al^19^ who compared the use of a digital caliper and the digital method by means of Bolton analysis. The authors did not find statistically significant differences between methods.

Other studies have also assessed the reliability of the digital method in relation to the manual one, namely: Oliveira et al,^4^ Mayers et al,^20^ Quimby et al,^1^ Bell, Ayoub and Siebert,^21^Zilberman, Huggare and Parikakis,^22^ Redlich et al,^23^ Veenema et al,^24^ Watanebe-Kanno et al.^25^ Although measurements obtained by the present study were not identical to those of the previous studies, the authors of the latter did not report any statistically significant differences between manual and digital methods, thereby corroborating the observations presented herein.

Santoro et al^15^ conducted a comparative study on the precision of measurements taken by the OrthoCAD system (Cadent, Carlstadt, NJ) on digital models and dental casts. Results showed that there were no statistically significant differences between measurements of overjet obtained by both methods, which agrees with the present study. However, there was statistically significant difference between methods, particularly with regard to teeth width and overbite. Bolton analysis requires teeth width measurements, which could affect total and anterior proportions. In the present study, there were no statistically significant differences for total or anterior proportion, which disagrees with Santoro et al.^15^

Bolton analysis did not result in excellent reproducibility, as demonstrated by Lin's concordance. One of the main reasons justifying the divergence between manual and digital methods is that points of reference may be challenging to locate and the opinion of the examiner regarding the exact location of a point can vary randomly.^26^While performing this study, it was found that measurements taken on the same tooth, with a minimum interlude time, presented minor divergences. This finding is similar to that reported by Shellhart et al^7^ who concluded that Bolton analysis can vary in + 2.2 mm when a bow divider is used. Tomassetti et al^12^ found that 72.7% of measurements decreased within 1.0 mm from one to another (0 to 2.8 mm) when Bolton analysis was calculated three times with a digital caliper on dental casts.

Even though Bolton analysis is widely diffused and relatively easy to apply, many practitioners do not use it for clinical evaluation, since the method is somewhat time consuming when the necessary calculations are performed.^12^ Digital measurements were easier to acquire than the manual ones, which is in agreement with studies by Abizadeh et al.^27^Tomasseti et al^12^concluded that the time spent by measuring casts with QuickCeph was 1.85 minutes, followed by Hamilton Arch Tooth System (HATS) at 3.4 minutes, OrthoCad at 5.37 minutes and caliper at 8.06 minutes. Mullen et al^19^also concluded that the digital method was faster than the manual one, thereby indicating an advantage in using the digital technique.

Additionally, computer programs assessing digital models, such as 3Shape^(r) ^Ortho Analyzer(tm) (Copenhagen, Denmark), which was used in this study, offer additional information beyond Bolton analysis, including: teeth size, overbite, overjet, analysis of models, symmetry and dental arch shape, intensity of the curves of Spee and Wilson, manufacture of orthodontic setup, among others. This is in accordance with the studies by Redmond,^14^Santoro et al^15^ and Tomassetti et al.^12^Quimby et al^1^ suggest that easy storage, manipulation of models and reduced measurement time are features that make the digital method more attractive to orthodontists.

## CONCLUSION

It is possible to conclude that Bolton analysis performed on tridimensional virtual models is as reliable as when it is performed on dental casts with satisfactory agreement.
